# CT brain image advancement for ICH diagnosis

**DOI:** 10.1049/htl.2018.5003

**Published:** 2019-12-10

**Authors:** Nor Shahirah Shaik Amir, Law Zhe Kang, Shahizon Azura Mukari, Ramesh Sahathevan, Kalaivani Chellappan

**Affiliations:** 1 Department of Electric, Electronics and System, Faculty of Engineering and Built Environment, Universiti Kebangsaan Malaysia, 43600 UKM Bangi, Selangor, Malaysia; 2 Department of Neurology and Radiology, Universiti Kebangsaan Malaysia Medical Centre, Jalan Yaacob Latif, Bandar Tun Razak, 56000 Cheras, Kuala Lumpur, Malaysia; 3 Department of Internal Medicine Services, Ballarat Base Hospital, Ballarat Health Services, Ballarat, Australia; 4 Faculty of Medicine, Dentistry and Health Sciences, University of Melbourne, Melbourne, Australia; 5 Florey Institute of Neuroscience and Mental Health, Melbourne, Australia

**Keywords:** image denoising, image segmentation, Wiener filters, computerised tomography, brain, medical image processing, image enhancement, CT brain image advancement, ICH diagnosis, primary intracerebral haemorrhage, computed tomography brain images, correct diagnosis, imaging modality, enhancement algorithm, CT images, CT brain images, final diagnosis, primary ICH, UKM Medical Centre, Digital Imaging, main sections, Wiener filter, wavelet, image enhancement, modified unsharp masking algorithm, UM algorithm, image analysis

## Abstract

A critical step in detection of primary intracerebral haemorrhage (ICH) is an accurate assessment of computed tomography (CT) brain images. The correct diagnosis relies on imaging modality and quality of acquired images. The authors present an enhancement algorithm which can improve the clarity of edges on CT images. About 40 samples of CT brain images with final diagnosis of primary ICH were obtained from the UKM Medical Centre in Digital Imaging and Communication in Medicine format. The images resized from 512 × 512 to 256 × 256 pixel resolution to reduce processing time. This Letter comprises of two main sections; the first is denoising using Wiener filter, non-local means and wavelet; the second section focuses on image enhancement using a modified unsharp masking (UM) algorithm to improve the visualisation of ICH. The combined approach of Wiener filter and modified UM algorithm outperforms other combinations with average values of mean square error, peak signal-to-noise ratio, variance and structural similarity index of 2.89, 31.72, 0.12 and 0.98, respectively. The reliability of proposed algorithm was evaluated by three blinded assessors which achieved a median score of 65%. This approach provides reliable validation for the proposed algorithm which has potential in improving image analysis.

## 1 Introduction

Intracerebral haemorrhage (ICH), an example of haemorrhagic stroke, is characterised by bleeding within the brain. ICH may be caused by hypertension, cerebral amyloid angiopathy (CAA), head trauma and arteriovenous malformation amongst others. Computed tomography (CT) is the main imaging modality used in diagnosing ICH during the initial evaluation of acute stroke [1]. The ability to detect haemorrhage is dependent on the quality of the CT brain images. Haemorrhage appears as a high-attenuation mass on CT scan but one of the limitations in ICH extraction is that the bleed may be poorly visualised on CT due to the presence of noise during the CT acquisition process [2].

**Fig. 1:**
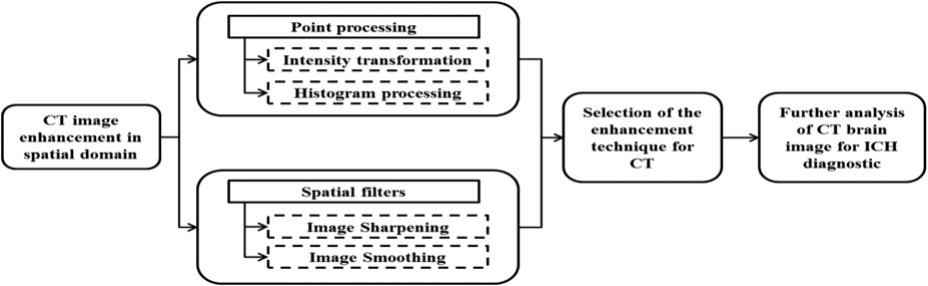
Block diagram of image enhancement techniques

Image processing and analytics play an important role in improving the visibility and interpretability of images. To ensure that the performance of ICH detection will be robust, irrespective of the quality of input CT images, it is essential to incorporate a CT enhancement algorithm in the haemorrhage extraction module. The basic block diagram for image enhancement categorisation [3–7] on CT is shown in Fig. 1.

Incorporating CT brain image advancement, which contains an aggregation of techniques, can improve the visual aspect of the CT image by reducing the noise and enhancing the contrast. This approach should facilitate clinicians in diagnosing ICH which in return substantially improves the healthcare diagnosis and treatment quality [8].

We propose to use an image advancement method to enhance the CT brain image. The proposed algorithm is a combination of denoising and an enhancement algorithm. We implemented Wiener filter, non-local means and wavelet to determine which are more effective in reducing Gaussian noise present in CT images [9–11]. To improve the visibility of the bleed, the edge was enhanced in the image using an unsharp masking (UM) algorithm. The edge of the haemorrhage is important in determining boundaries of the bleed within the CT brain image which works by detecting discontinuities based on the image contrast [12]. The reliability of the algorithm was assessed by three blinded assessors to determine the best advancement technique for CT images.

## 2 Related work

The conventional visualisation uses windowing in the neighbouring pixels in an image, which is known as contrast enhancement [13]. Ji's adaptive enhancement technique incorporative of local image contrast and just-noticeable-difference (JND) is an improvised visualisation algorithm [14]. This adaptive technique obtains a structured image from the original as practised in UM with an adaptive contrast gain. The adaptive contrast gain is determined by addressing the function of spatial activity for visibility of noise and function of spatial frequency that represents the threshold contrast. Adaptive contrast gain technique facilitates the selection of a higher gain of local contrast against the threshold value in ensuring more reliable low contrast object detection in an image. The algorithm reduces the ringing artefact and enhances the edges of the image. Even though the adaptive gain technique enhances the visualisation, the manual selection of the contrast gain limits the automation of this algorithm.

Medical image visualisation enhancement has been furthered by a novel digital radiographic image processing system, which includes the property of scalability and adaptability introduced by Yin *et al.* [15]. The ability of this algorithm is using scalable edge enhancement in identifying subtle structure increased medical image quality in clinical diagnosis. The advantage of the proposed algorithm is the scalability in edge kernel operator selection which bridges the gap in the conventional methods. The adjustable degree of edge enhancement provides flexibility in selecting the kernel size based on the selected images. This approach allowed a small-sized kernel operator selection from a low-resolution version of the image to extract the edge information that reduces the computation time. The extracted information were scaled up to the original image size based on a scalable factor (0.0–2.0) and fixing weight factor at 2 to prevent additional noise, then added to the original image, making it similar to the conventional UM algorithm approach. The proposed method was evaluated against an UM algorithm using histogram and subjective assessment of five blinded assessors. The small-sized scale factor selection ability in adjusting the degree of edge enhancement to reduce the computational time, as well as suppressing the noise places this algorithm superior to the previous scale factor selection method. Although only a single convolution computation is required, it is possible that fine details of information from the image might be lost during sub-sampling of the image into a smaller size.

Almost a decade later, Ahmed and Nordin [16] improved the diagnostic visualisation of medical images using a denoising and enhancement algorithm. In this algorithm, the visualisation enhancement included two additional steps: use of a median filter for denoising and contrast limited adaptive histogram equalisation for overall image enhancement, in addition to sharpening the edges, as the previous researchers. This Letter demonstrated that using a median filter was able to remove the impulse noise from the medical images and the classic UM controls flexibility by adjusting the parameters. Nevertheless, impulse noise is not present in all medical images. Different image modalities may contain different types of noises. The classic UM algorithm introduces more noise if it is applied to the CT images, though the parameters can be adjusted to enhance the edges.

The medical image visualisation started moving toward specific disease diagnosis such as stroke. Tan *et al.* [17] enhanced soft tissue areas in CT brain images to improve ischaemic stroke detection using extreme level, eliminating brightness preserve bi-histogram equalisation. The outcome of their research highlighted that contrast of the soft tissues’ area is enhanced, and the background brightness of the image is preserved better than the histogram equalisation. However, dualistic sub-image histogram equalisation outperformed the proposed technique. In spite of that, these two techniques still caused over-enhancement in the image background that leads to blurry edges.

There was an effort by Anand and Selva Kumari [18] at improving the blurry edges by applying an isotropic hyperbolic secant square filter. This technique was approached by convolving image data with the matrix representation of coefficient finite impulse response filters to reduce the noise and enhance edge in the CT images. Hence, the outcome was subjective and not evaluated by the standard image quality assessment.

The edge enhancement trail of CT images was achieved using an algorithm to reduce noise sensitivity in enhancing the edges in the image using various techniques. There is no specific research on image enhancement that focuses on ICH, in particular, edge enhancement, aimed at possibly differentiating types of haemorrhage [19].

## 3 Methodology

About 40 CT brain image datasets were collected in Digital Imaging and Communication in Medicine format from UKM Medical Centre. Patients with primary ICH (p-ICH) were selected, and any secondary cause of ICH was excluded. According to Sahni, the incidence of p-ICH is more common which accounts for 80% of deaths [20]. Risk factors of p-ICH include hypertension and CAA. We exclude any secondary ICH in this Letter consisting of trauma, subarachnoid haemorrhage, primary or secondary tumour as the mechanism, location and appearance of haemorrhage in these conditions is less uniform, and they may overlap the p-ICH bleeds which may appear as microbleed on CT scan. Patients who did not undergo magnetic resonance imaging (MRI) were also excluded.

### 3.1 Digital data acquisition

All the patients diagnosed with ICH were identified from the radiology department database. A manual search was conducted to identify patients with p-ICH and those patients who had undergone CT and MRI in the same year were included for analyses. The images were anonymised, apart from the sex and age. All CT images were resized from 512 × 512 to 256 × 256 px^2^ resolution. The resized images were processed using the image advancement techniques which incorporated the denoising and enhancement methods.

### 3.2 Image processing advancement

Image processing advancement aids in improving visuals and visibility of the image. This Letter highlights the possible enhancement techniques that can be selected to sharpen and smooth the CT brain image. The method is divided into two sections. The first part is regarding the best filter that can be used to reduce the noise present on CT brain images while the second part highlights the optimal filter for edge enhancement.

#### 3.2.1 Denoising in CT images

**Fig. 2:**
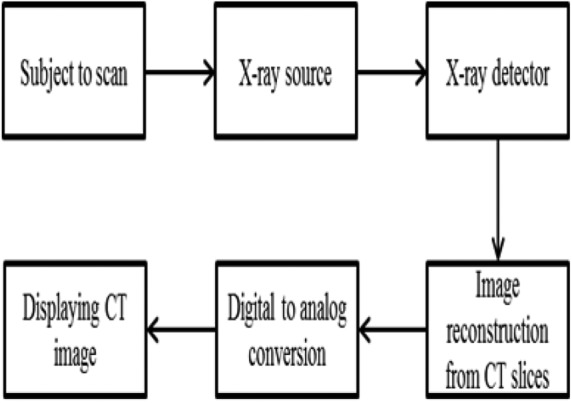
Block diagram of producing a CT image

Noise present in CT brain images is due to irregular fluctuations of pixel intensities during the digital data acquisition process. The unwanted fluctuations produced tend to obscure the CT images. The common noise existing in CT images is Gaussian noise, also known as the electronic noise, which is introduced by the X-ray detectors during acquisition process from CT scan. Gaussian noise adds grainy appearances to the image, which may deteriorate fundamental information of the size, shape and the pixel intensity values or texture present in the region of interest. The process of obtaining a CT image is shown in Fig. 2.

Three different filters were applied to the images for noise reduction, which consists of Wiener filter, non-local means and wavelet denoising. The benefits and limitations of the filters are detailed in Table 1 to evaluate the optimal noise reduction algorithm for CT images.

**Table 1:** Evaluation of noise reduction techniques

Noise removal filters	Advantages	Drawbacks
wiener filter	reduces additive white Gaussian noise effectively	requires longer computational time
non-local means	noise is reduced better compared with the mean filter	it disrupts the structure of the image during denoising
wavelet	image reconstruction after the denoising during decomposition level experience little loss of information	manual identification of decomposition level varies for each image

#### 3.2.2 CT image enhancement for ICH diagnosis

*Image sharpening using the conventional UM method:* UM algorithm sharpens an image by removing the low-level frequency spatial information from the original image. It highlights the edges of the image and increases the intensity of image in darker regions compared with lighter ones [21]. The image will first be blurred using a low-pass filter and subtracted from the original image-producing lines of edges from the image known as an unsharp mask. An additional operation is performed between the unsharp mask and original image forming a sharper image [22] via the equations below:

(1) $$g\left({x\comma \; \, y} \right)= f\left({x\comma \; \, y} \right)- f_{{\rm smooth}}\left({x\comma \; \, y} \right)\eqno\lpar 1\rpar $$

where *f*(*x*, *y*) is the input image, *f*_smooth_ (*x*, *y*) is the blurred version of the input image and *g*(*x*, *y*) is the processed image. The complete UM operator is defined as below:

(2) $$f_{{\rm sharp}}\left({x\comma \; \, y} \right)= f\left({x\comma \; \, y} \right)+ k\left[{g\left({x\comma \; \, y} \right)} \right]\eqno\lpar 2\rpar $$

where *k* is a scaling constant which varies between 0.2 and 0.7 with the larger values producing a sharper image. The limitation of the conventional UM algorithm is that though the edges of the image are enhanced, it introduces more noise in the image as well.

**Fig. 3:**
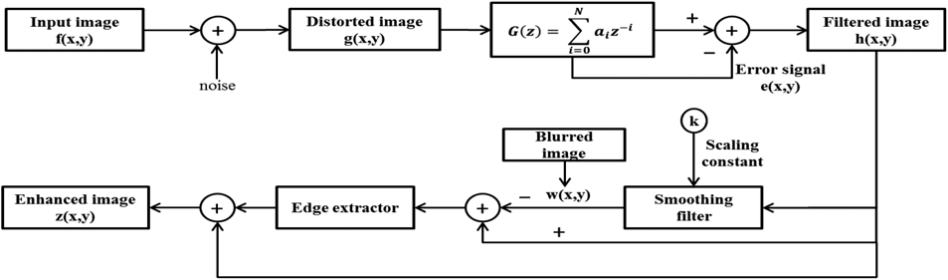
Block diagram of the modified UM algorithm

*Image sharpening using the modified UM method:* We propose to improve the conventional UM method by introducing a *k* scaling constant at the smoothing filter, as shown in Fig. 3. Gaussian noise is introduced in the input image and denoised using a smoothing filter. The denoised image is smooth with the scaling constant *k* varied from 0.1 to 0.4 but the mean square error (MSE) of the images average at 0.4. Any value higher or lower than this will deteriorate the spatial information of the image. The blurred image produced from the smoothing filter is combined with the denoised image to extract the low-level features. The enhanced image is formed through a combination of the denoised image with the extracted edges image. This modified UM algorithm is expected to enhance the CT brain image without introducing more noise into it.

## 4 Results

### 4.1 Denoising techniques in CT brain images for ICH diagnosis

The evaluation of all the three methods was based on MSE, peak signal-to-noise ratio (PSNR) and variance and structural similarity index (SSIM) [23]. This test was done to identify the best noise reduction technique on CT brain image. The results are shown in Table 2. Among the three denoising techniques, Wiener filter resulted in the highest PSNR (25.31). Both Wiener and wavelet have the same variance of 0.09.

**Table 2:** Evaluation of denoising techniques on CT brain images

Number of CT image	Denoising techniques											
	Wiener filter				Non-local means				Wavelet			
	MSE	PSNR	Variance	SSIM	MSE	PSNR	Variance	SSIM	MSE	PSNR	Variance	SSIM
average median of 40 images	1.30	25.31	0.09	0.44	0.01	22.88	0.09	0.40	1.56	24.47	3.96	0.42

### 4.2 CT image enhancement methods for ICH diagnosis

The three different denoised images were further processed using a modified UM algorithm for edge enhancement. This technique outcome was evaluated using MSE and SSIM. The lowest MSE obtained in non-local means (NLM) and highest SSIM in Wiener filter.

**Fig. 4:**
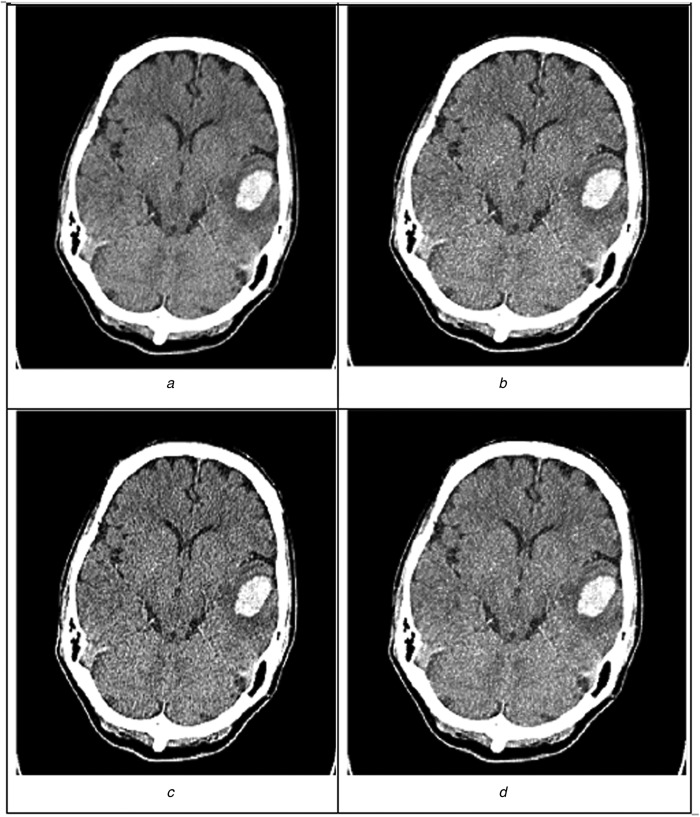
Image showing the image advancement techniques implemented on CT image *a* Original grey-scale image *b* Wiener filter with modified UM *c* Non-local means with modified UM *d* Image denoised with a wavelet and enhanced with modified UM

**Fig. 5:**
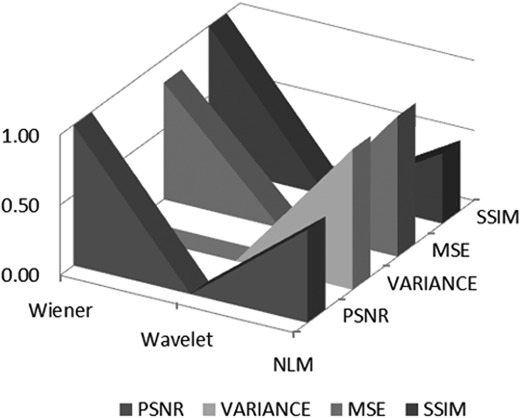
Denoising techniques applied to CT brain images: Wiener, wavelet and NLM

**Fig. 6:**
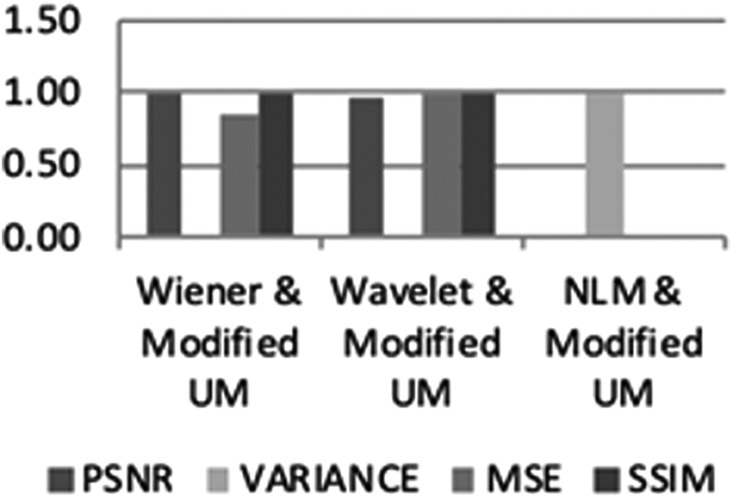
Enhancement of denoised CT brain images using a modified UM algorithm for ICH classification

The evaluation of all 40 samples for patients with p-ICH is shown in Table 3. A sample of edge-enhanced image for all the three denoising techniques is represented in Fig. 4. Denoising using Wiener and enhancement with modified UM algorithm outperforms the other two combinations in enhancing the edges in CT brain images by depicting the lower MSE, higher PSNR, variance and SSIM values as in Figs. 5 and 6.

**Table 3:** Evaluation of CT image edge enhancement techniques

Number of CT image	Edge enhancement techniques											
	Wiener filter + modified UM				Non-local means + modified UM				Wavelet + modified UM			
	MSE	PSNR	Variance	SSIM	MSE	PSNR	Variance	SSIM	MSE	PSNR	Variance	SSIM
average median of 40 images	2.89	31.72	0.12	0.98	0.02	16.78	0.14	0.06	3.38	31.05	0.12	0.97

Each blinded assessor was given a set of CT brain images corresponding to 40 patients. Each set consisted of three processed images using the combination of denoising and enhancement techniques. All the three blinded assessors were required to rate the most enhanced edges of ICH bleed on CT brain images based on their visual perspective.

**Fig. 7:**
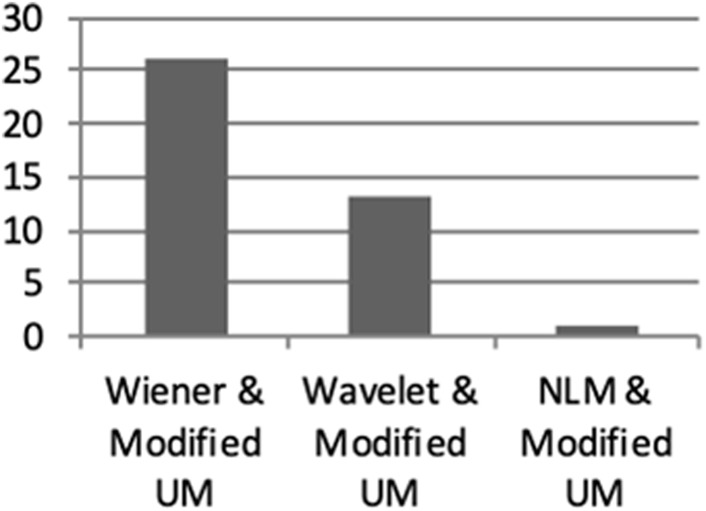
Average median score is given by all three blinded assessors based on 40 sets of CT images with the combination of denoising and enhancement techniques

Results obtained from the three blinded assessors were analysed to identify the best technique for denoising and enhancement to be used on CT brain image for ICH classification. The median score for all the 40 sets of the processed CT images was computed and plotted as shown in Fig. 7. From the assessment, it is clear that the proposed method of using Wiener filter with the modified UM algorithm is more preferred compared with the other two methods applied on CT images. The proposed technique is expected to support the neurologist to examine ICH CT images more accurately.

## 5 Discussion

In this Letter, we assessed an image advancement technique to improve CT images of a brain haemorrhage. This method is used as the basis to further analyse CT brain image in computer vision. From a technical perspective, incorporation of image advancement techniques in computer vision will allow better visualisation of low-level features and remove irregular fluctuations of pixel intensities present in the image. This advanced computer vision, which leverages image advancement techniques, strengthens the computer's ability to process the image with multiple levels of abstraction. The capability of visualisation allows extensive exploration of the low-level features representing the CT brain structure. Through this approach, the processed CT image is expected to assist clinicians in improved diagnosis of ICH in the absence of MRI.

In our Letter, only a single CT slice for each patient was processed. The decision not to use multiple slices was based on the advice of the clinicians and criteria defined for slice selection considering the option of cloud-based storage and timely processing for remote areas. In exploring image enhancement, three different filters were selected for denoising: Wiener filter, non-local means and wavelet. The selected filters have been extensively used in CT image processing by past researchers [9–11].

Wiener filter is more consistent in removing the Gaussian noise present in CT images. It produces the desired signal for image restoration based on linear time-invariant filtering of an observed noisy image. In other words, it provides the optimal trade-off between inverse filtering and noise smoothing, resulting in the minimum MSE value of the denoised image [24].

Non-local means provide a less blurred image, based on its property to calculate the mean for the whole image, compared with mean filter, which is dependent on the neighbouring pixels [25]. On the basis of the result, we can observe that though the image is smooth, the fine details and structure of the image were not preserved.

Wavelet denoising is different compared with the classic filters, whereby it attempts to remove the noise while preserving the spatial information regardless of its frequency content. It consists of three main steps: (i) linear forward wavelet transform, (ii) nonlinear thresholding and (iii) linear inverse wavelet transform [26]. The principle of thresholding and shrinkage plays an important role in wavelet denoising in two-dimensional images. Initially, the image is decomposed into 14 levels. The value was selected based on trial-and-error approach of a range from 0 to 50. Level 14 was the finest frequency resolution that was used to achieve a denoised image. In the next stage of wavelet transform, we deal with the wavelet coefficient which represents the image features. A low wavelet coefficient value indicates noise, and we shrink or remove the noise without distorting the image quality. Soft thresholding was applied, and the data was reconstructed using the inverse wavelet transform. However, the main limitation here is the selection for the right decomposition value that varies between each image for different patients.

The standard image quality assessment for noise reduction is based on the measurement of MSE, PSNR, variance and SSIM. On the basis of previous studies, PSNR is the best performance evaluation to measure the most denoised images [27, 28]. The high PSNR value achieved from the Wiener filter indicates that more Gaussian noise was removed from the CT brain images of ICH. Also, the adaptive nature of Wiener filter makes it a better option to be used in removing the Gaussian noise in CT images.

UM is one of the techniques optimally used for edge enhancement [19, 29, 30]. However, the main limitation is that it introduces noise while sharpening the image leading to more deterioration [29]. The modified UM improves this limitation by enhancing the role of the smoothing filter in the algorithm. On the basis of the result, it shows that the contribution of parameters in decision making for edge enhancement relies heavily on the MSE and SSIM values. Lower MSE value indicates the reduced error present from the mean of neighbouring pixels, while least variance value represents a lower risk of error present in the image after enhancement was applied. The higher SSIM value depicts the lower distortion of the luminosity, contrast and image structure. The selection approach was based on the parametric measurements for all three techniques from Figs. 5 and 6. These comply with the three blinded assessment results for different patients in Fig. 7.

We propose to apply the image advancement method in enhancing the CT brain image for ICH. The method starts by denoising the CT image using the Wiener filter, non-local means and wavelet denoising algorithm followed by the use of the modified UM algorithm to enhance the edges of the image. In our Letter, denoising using Wiener filter provided the best denoised image for enhancement, using the modified UM algorithm, and resulted in an image with the least amount of noise and error. There is potential to make this algorithm available in cloud image analytics which will allow CT users to have enhanced images to aid in diagnosis.

## 6 Conclusion

The main objective of this Letter was to reduce noise and improve the visibility of ICH bleed on CT brain images, without reducing their spatial information. The proposed method can reduce noise present in CT images and enhance the edges to visualise the bleed better. The result of this Letter has provided a new opening to explore further different bleed categories in CT images for ICH classification. A more precise and advanced filtering technique may provide sensitive image analytic algorithms that can employ a deep learning approach and provide more reliable classification in ICH screening and diagnosis.
